# Wounds by Small Projectiles

**Published:** 1898-05-21

**Authors:** 


### WOUNDS BY SMALL PROJECTILES.

The rumours of war which abound on every side lend great interest to all that has to do with bullets. Now Surgeon-Major-General Hamilton has had much to do with these missiles, not only in his own professional capacity but also as a sportsman, and what he says in the *British Medical Journal* about the various modern projectiles and their effects is worthy of careful notice.

Without going through the whole history of the evolution of the dum-dum bullet, it may be pointed out that when rifles came into general use in our army they were of comparatively large bore, the Snider sending a bullet of 0·577 in. diameter, and that, as by successive steps the bore was diminished and the velocity was increased, the bullets tended more and more to pass through the body without producing such an amount of injury as to stop a savage and determined foe, unless some vital organ or some important bone were hit. "In European warfare," we are told "this was of comparatively little consequence, as civilised man is much more susceptible to injury than savages. As a rule, when a 'white man' is wounded he has had enough, and is quite ready to drop out of the ranks and go to the rear; but the savage, like the tiger, is not so impressionable, and will go on fighting even when desperately wounded."

Even the Martini-Henry, with its hardened projectile of 0·450 diameter, was complained of in this respect, but when in the Lee-Metford the diameter of the bullet was reduced to 0·3 in., its inefficiency as a "stopper" became too obvious to be passed over. If a bone was hit it was comminuted to a remarkable degree, but if the soft parts only were involved very little damage might be done. There is the well-known instance of the "Chitrallee," who walked into one of our field hospitals to have his wounds dressed, when it was found that no fever than five Lee-Metford bullets had passed through various parts of his body. The men felt that their weapons failed in "stopping" power, and that though they might hold straight and hit their adversary fairly, he was still able to charge home, and perhaps cut them down. Hence the invention of the dum-dum, which, by expanding, makes a larger wound. It must be understood, however, that severe as is the wound that it produces, this is in no sense a new and savage form of injury peculiar to this particular form of bullet. What has been done is merely to make the new little bullet make a wound in some degree approaching that caused by the old big one. It is "in no sense an 'explosive bullet,' but merely a partially expanding one; and even now is not for a moment to be compared in its destructive powers to the Snider bullet, which was never objected to by the Geneva Convention or the Great Powers."

